# Some More Quackery and Bogus Diplomas*This was set up for August, but crowded out; meantime it appeared in N. Y. Medical Record, with editorial comments.—Ed.

**Published:** 1896-10

**Authors:** 


*Some More Quackery and Bogus Diplomas.—From all parts of Texas subscribers are sending the "Red Back" evidences of quackery, quack advertisements, and resorts of a certain set to get practice, as evidence of what the educated physician has to contend with. The people are gullible, and these sharp and unscrupulous fellows are taking the bread and butter out of the mouths of the deserving physicians. From Bonham comes a pumpkin colored dodger headed "Persecution," and signed "O. Robertson, M. D., Steam Doctor, Bonham, Texas, specialist for chronic disease." He alleges that some one has threatened him, and makes it pretext to lay claim to sympathy. The document is a remarkable mixture of Billingsgate and "whine," and is not worthy of notice. If this form of "practitioner" cannot be gotten at in some way, more the pity; it illustrates forcibly the inefficiency of our medical laws.

Still Another:—This one illustrates the defects in the law governing charters. It also emphasizes the necessity of concert

*This was set up for August, but crowded out; meantime it appeared in N. Y. Medical Record, with editorial comments.—Ed.

of action on the part of State Boards of Health looking to a discriminating method of classifying diplomas. Here is a bid to those who have "all the equipments of a doctor except a diploma" (*vide supra*) "to graduate" in short time and at little cost. This letter was sent to one of the JOURNAL's subscribers, a prominent physician known to us to be a graduate of Jefferson Medical College, and a member of the State Medical Association. Is there any way to suppress these "mills?"

Here is the letter:

DEAR DOCTOR:—We notice your name in a Medical and Surgical Directory, but with a * appended. This usually means (although not necessarily so), that the person so designated is not a graduate of a medical school, and has no diploma. If, however, it should be that you are a graduate, and have a regular diploma, then we can but tender our most sincere apologies for troubling you on the matter. But, on the other hand, if you are not a graduate, and have no regular diploma, then the perusal of the enclosed prospectus cannot fail to be of the most primary importance and interest to you. We would also desire to draw attention to the fact, that to practicing physicians our fees are much reduced from the regular rate. To this class our fees are $35.00, all inclusive.

As proof of our legal standing to confer the degree of M. D., we can supply certified copies of our charter at 25 cents each, simply covering the cost of certifying officer's fee.

Trusting soon to hear from you, and standing ready to answer any or all question you may wish to submit.

We are yours very sincerely,

WISCONSIN ECLECTIC MEDICAL COLLEGE.

This "college" does not make attendance even, a requisite to graduation! It is "regularly chartered."—Ed.

